# Influenza vaccination coverage in elderly and high-risk adults: characterization of associated factors

**DOI:** 10.31744/einstein_journal/2021AO5830

**Published:** 2021-06-28

**Authors:** Kevin Francisco Durigon Meneghini, Camila Furtado Hood, Letícia Oliveira de Menezes, Raúl Andrés Mendoza-Sassi, Samuel Carvalho Dumith

**Affiliations:** 1 Faculdade de Medicina Universidade Federal do Rio Grande Rio GrandeRS Brazil Faculdade de Medicina, Universidade Federal do Rio Grande, Rio Grande, RS, Brazil.; 2 Faculdade de Medicina Universidade Católica de Pelotas PelotasRS Brazil Faculdade de Medicina, Universidade Católica de Pelotas, Pelotas, RS, Brazil.

**Keywords:** Influenza vaccines, Barriers to access of health services, Health services research, Primary prevention, Public Health, Risk groups

## Abstract

**Objective:**

To evaluate the prevalence and factors associated with non-vaccination against influenza in the risk group.

**Methods:**

A cross-sectional, population-based study, carried out in the city of Rio Grande (RS). The outcome was defined as belonging to risk groups and not having been vaccinated in the last 12 months. Demographic, socioeconomic, behavioral variables, and access for health services were analyzed.

**Results:**

In this study, 680 individuals participated. The prevalence was 46.0% (95%CI: 41.8-50.3), ranging from 27.9% (elderly) to 81.8% (pregnant women). Young adults, single, intermediate socioeconomic bracket, smoker, with depressive symptoms, who did not perform physical activity and did not consult a physician in the last year, had a higher prevalence of non-vaccination.

**Conclusion:**

Half of the sample was not vaccinated in the period. Due to the similarity of influenza-like illness and the coronavirus 2019 disease (COVID-19), increasing vaccination would minimize mortality and use of hospital beds due to influenza, optimizing the response of hospital capacity.

## INTRODUCTION

Vaccine, from Latin *vaccine*, means “derived from cow”. Edward Jenner was responsible for the records in the 18^th^ century. He observed that peasants were not affected by smallpox when in contact with infected cows,^(1,2)^which was recognized when the efficacy of the microorganism inoculation was verified in the immunological stimulation, with a lower number of people getting sick.^(1)^

In Brazil, the first vaccination campaign, conceived by Osvaldo Cruz, took place in 1904 and focused on reducing smallpox morbidity and mortality.^(1,2)^ However, with the fear of the unknown and the population’s misunderstanding, popular resistance was triggered, culminating in the revocation of compulsory vaccination and the failure of the campaign. This became known as the Vaccine Uprising.^(1-3)^

With better understanding of the population about the benefits of vaccination, the habit of vaccination was gradually created in the population and, due to the success achieved, and became compulsory approximately 50 years ago. Today, 20 vaccines are mandatory, aiming to protect people from controlled agents, which have already been responsible for decimating populations.^(3)^

The World Health Organization (WHO) is one of the agencies responsible for developing and researching vaccines, with the purpose of eradicating diseases, such as smallpox, poliomyelitis, and varicella.^(2)^ In Brazil, the agency responsible for the vaccination schedule and for measuring compliance with it is the Ministry of Health, through the National Immunization Program, adapting, within the territory, the most specific vaccination for each region.^(2,3)^ The National Immunization Program does not operate exclusively in Brazil; it is an international reference and partner of other countries, such as East Timor, Palestine, and the West Bank.^(2)^

In 1999, the first vaccination campaign against influenza aimed to reduce morbidity and mortality in the elderly due to their vulnerability, reaching 87.34% coverage, and exceeding the goal by 17.34%.^(3)^ After this success, this vaccine has been offered throughout the year and is part of the annual vaccination calendar.^(3)^ The vaccination goal increased according to the success of vaccination coverage and the demographic change demonstrated by the Brazilian Institute of Geography and Statistics (IBGE - *Instituto Brasileiro de Geografia e Estatística*)- from 80% in 2008, and 90% in 2017,^(4)^covering between 89% and 92%.^(5)^

In 2020, Brazil was in its 22^nd^ influenza vaccination campaign, but with a different outlook: a new pandemic, the coronavirus 2019 disease (COVID-19) made the world and Brazil more attentive to influenza symptoms.^(4,6)^ Vaccine coverage in that year aimed mainly to facilitate the differential diagnosis between the common influenza syndrome and COVID-19, allowing a more accurate investigation and diagnosis, and reducing disease morbidity and mortality.^(4,6)^

Currently, the most vulnerable groups to infection, temporary immunosuppression, or risk of decompensation of the underlying disease are given priority.^(7)^ Among these, in addition to the elderly, are children aged between 6 months and 5 years; pregnant and postpartum women; healthcare and prison workers; indigenous peoples; population deprived of freedom; obese individuals; patients with chronic non-communicable diseases (NCD), such as respiratory, cardiac, renal, hepatic, neurological conditions, and diabetes; patients with drug-induced or congenital immunosuppression; and trisomy carriers.^(4)^

In 2019, the vaccination coverage of risk group members exceeded 84%.^(4)^ Pregnant women and children had the lowest coverage (84%), the only ones not reaching the goal.^(4)^ The elderly had vaccination coverage of roughly 99%.^(4)^ Other prioritized diseases accounted for 17% of doses applied in the campaign; among them, patients with chronic respiratory diseases, chronic heart diseases, and diabetes were responsible for 81% of doses within the group.^(4)^

Although it seems harmless, the influenza-like illness can present with severe signs and symptoms, and can progress to death.^(4,7)^ Depending on how the pathogen is introduced into the population, devastating consequences can occur, as in the 2009 pandemics of H1N1,^(8,9)^ and currently, of COVID-19.^(6)^ The official statistics published until July 2020 reported a mortality rate of 3.9% in Brazil.^(10)^ When healthy persons are infected, they are more likely to present with mild symptoms or be asymptomatic. However, individuals with an existing condition or a debilitating health status have a higher likelihood of being severely affected by the disease.^(7)^

In Brazil, respiratory diseases are frequent causes of hospitalization (nearly 640 thousand admissions in 2019), especially in the elderly population. Even with an average stay of 6 days, many demanded prolonged hospitalizations, beds in intensive care unit (ICU), and sometimes evolved to death. In 2019, the mortality rate was 9.45 per 100 thousand inhabitants.^(4,7,11)^

Some people become unprotected, whether included or not in the priority group,^(4)^ and some reasons for not vaccinating are fear, belief that it does not work, perception of a low risk of disease, unpleasant experience with previous immunizations, lack of knowledge, and not understanding the provision by the Unified Health System (SUS - *Sistema Único de Saúde*).^(12)^ The vaccination campaign has an educational nature, since it introduces general knowledge about influenza, explains the importance of the act, demonstrates possible morbidity and mortality of the disease, and promotes population immunization.^(3,4)^

Even with the high coverage rates presented by the campaign, population surveys that contribute to the official data are needed, aiming to identify factors (socioeconomic, demographic, lifestyle, comorbidities, proximity to the Primary Healthcare Unit, and registration of users) associated with non-vaccination, to assist health managers in identifying characteristics that require greater attention, so that the goal of vaccination coverage is achieved.^(12)^

## OBJECTIVE

To evaluate the prevalence and factors associated with non-vaccination against influenza in at-risk groups.

## METHODS

The city of Rio Grande is located in the state of Rio Grande do Sul, in the extreme south of Brazil, with approximately 200 thousand inhabitants, 95% living in the urban area. Its economy is mainly based on port activities. The city Human Development Index (HDI) is 0.744 and the Gross Domestic Product (GDP) *per capita* is R$ 36,816.67.^(13)^ In addition, the city has two hospitals, one of which is totally dedicated to SUS, and 32 Primary Healthcare Unit.

This study derived from the project *Saúde da População Rio Grandina*, (Health of Rio Grande population), which investigated diseases and risk factors in individuals aged over 18 years, in the urban area of Rio Grande, excluding those institutionalized in nursing homes, hospitals, prisons, and those with physical and/or cognitive disabilities that prevented them from answering the questionnaire. Data was collected in 2016. More methodological details can be found in an earlier publication.^(13)^

Two sample calculations were performed. The first checked the prevalence of the outcomes, and the second, factors associated with the outcomes. The sampling process was composed of two stages: first the census sectors, followed by the households. We carried out a systematic selection, choosing 72 out of 293 eligible census sectors (25%), ten households per sector on average, selecting more sectors, and fewer households, minimizing the design effect.

The outcome was considered as not having received an influenza vaccine in the 12 months prior to the survey. Only individuals who were in risk groups according to the Ministry of Health were considered in the denominator:^(4)^ the elderly; pregnant women; patients with chronic NCD (respiratory, cardiac, renal, diabetes mellitus type 1 or type 2 on medication, and obesity grade III), and indigenous people. The independent variables included were sex (male or female), age group (in years), skin color (white or others), marital status (married, single, or widowed/separated), education (in years), asset index (in terciles), smoking (non-smoker and current smoker), excessive alcohol consumption (yes or no), physical activity (yes or no), food insecurity (yes or no), level of stress (in terciles), self-perception of health status (excellent/very good, good, fair/poor), depressive symptoms (yes or no), having health insurance (yes or no), having seen a doctor in the last 12 months (yes or no), having been visited by a community health worker (yes or no/does not know), and household registered in the neighborhood Primary Healthcare Unit (yes or no/does not know).

The asset index was obtained by analysis of the main components on a 11-item list of household assets or characteristics. The first component that explained 30% of variance in all variables (eigenvalue of 3.3) was extracted and divided into terciles. Excessive alcohol consumption was defined as consuming five or more drinks for men and four or more drinks for women in the past month.^(14)^ Physical activity was measured using the leisure-time section of the International Physical Activity Questionnaire^(15)^ and defined as inactive (no activity) or somewhat active. Food insecurity (lack of availability and access to food) was measured using the Brazilian Food Insecurity Scale.^(16)^ Stress was measured by the Perceived Stress Scale,^(17)^ and depressive symptoms were obtained using the Patient Health Questionnaire-9 (PHQ-9).^18^

Concomitant to data collection, a 10.5% partial reproduction of the instrument was performed for data quality control (Kappa index of 0.80). The questionnaires were coded, reviewed, and double typed using the EpiData program, version 3.1, and the data were later transferred to the Stata statistical package, version 11.2, performing exploratory analysis of the database, transformation, and categorization of the variables.

We started with a descriptive analysis, describing the absolute and relative frequencies of the variables. Bivariate and multivariable analysis was performed using Poisson regression, considering the effect of sample design as 1.3. The multivariable analysis was done in levels, controlling the variables for those of the same level or levels above. The variables were divided into four levels: first, demographic and socioeconomic; second, behavioral; third, health; and fourth, health services. Wald test for heterogeneity was used, maintaining the adjusted model variables with a p-value of ≤0.20. The level of statistical significance was set at 5% for two-tailed tests.

The project was approved by the Health Research Ethics Committee of the *Universidade Federal do Rio Grande* (FURG) (CAAE: 52939016.0.0000.5324). Participants signed the Informed Consent Form (ICF) and, if illiterate, consented by fingerprint after the form was read aloud.

## RESULTS

The sample was composed of 680 individuals (90% response rate). The mean age was 55 years (standard deviation - SD =17), ranging from 18 to 96 years. In the sample, 59.3% were female; 52% had zero to 8 years of schooling; 45.9% were 60 years or older; 84.3% reported being white, and 44% were married. Smokers were 17.5%, those who consumed alcohol in excess 9%, approximately 69% did not perform physical activity; 33.5% had food insecurity, 45% perceived their health as regular or bad, and 29.7% had depressive symptoms; 47.5% had no health insurance, 17.4% had not seen a physician in the last year, and 23.5% reported having received a visit from a community health worker at home, while 34.9% reported a home registered at a Primary Healthcare Unit (Table 1).

**Table 1:** Characteristics of the sample comprising the risk groups for vaccination against influenza

Variable	n (%)
Sex	
Male	277 (40.7)
Female	403 (59.3)
Age range, years	
18-39	145 (21.3)
40-59	223 (32.8)
≥60	312 (45.9)
Skin color	
White	573 (84.3)
Others	107 (15.7)
Marital status	
Married	299 (44.0)
Single	211 (31.0)
Widowed, separated	170 (25.0)
Schooling, years	
0-8	353 (52.0)
9-11	197 (29.0)
≥12	129 (19.0)
Asset index	
Poorer	246 (36.2)
Intermediate	203 (29.9)
Richer	230 (33.9)
Smoking	
No	561 (82.5)
Yes	119 (17.5)
Excessive alcohol consumption	
No	617 (91.0)
Yes	61 (9.0)
Physical activity	
No	470 (69.3)
Yes	208 (30.7)
Food insecurity	
No	452 (66.5)
Yes	228 (33.5)
Stress, terciles	
Least stressed	245 (36.2)
Intermediate	230 (33.9)
Most stressed	203 (29.9)
Perception of health	
Excellent/Very good	104 (15.3)
Good	270 (39.7)
Regular/Poor	306 (45.0)
Depressive symptoms	
No	477 (70.3)
Yes	201 (29.7)
Health insurance	
No	323 (47.5)
Yes	357 (52.5)
Consultation with physician	
No	118 (17.4)
Yes	562 (82.6)
Community health worker	
No/does not know	520 (76.5)
Yes	160 (23.5)
PHU in the neighborhood	
No/does not know	442 (65.1)
Yes	237 (34.9)

PHU: Primary Health Unit.

On table 2, the risk groups were separated, in descending order, according to the outcome.

**Table 2:** Description of risk groups and prevalence of non-vaccination

Risk group	n (%)	Prevalence of non-vaccination (%)
Pregnant women	11 (1.6)	81.8
Ischemia (stroke)	38 (5.6)	56.8
Chronic respiratory disease	201 (30.0)	52.2
Cardiopathy	133 (19.5)	47.0
Morbid obesity	13 (2.0)	46.2
Hypertension	366 (53.6)	44.4
Cancer	37 (5.4)	43.2
Diabetes	90 (13.2)	41.1
Renal failure	34 (5.0)	36.4
Elderly	315 (46.1)	27.9
Total	680 (100.0)	46.0

The prevalence of individuals in the risk group not immunized in the last 12 months was 46.0% (95% confidence interval - 95%CI: 41.8-50.3), ranging from 27.9% (elderly) to 65.3% (had not seen a physician in the last year), and 65.5% for individuals aged 18 to 39 years (Table 3).

**Table 3:** Crude and adjusted analysis of the prevalence of non-vaccination against influenza for risk groups

Variable	Prevalence (%)	Crude analysis PR (95%CI)	Adjusted analysis PR (95%CI)
Sex		p=0.63	p=0.68
Male	46.9	1.00	1.00
Female	45.4	1.03 (0.90-1.18)	1.03 (0.90-1.18)
Age range, years		p<0.01	p<0.01
18-39	65.5	2.34 (1.84-3.00)	2.06 (1.57-2.69)
40-59	58.7	2.11 (1.66-2.67)	2.03 (1.60-2.58)
≥60	27.9	1.00	1.00
Skin color		p=0.95	p=0.52
White	46.1	1.00	1.00
Others	45.8	0.99 (0.81-1.23)	0.93 (0.75-1.16)
Marital status		p<0.01	p=0.12
Married	40.5	1.00	1.00
Single	62.1	1.53 (1.26-1.86)	1.24 (1.01-1.52)
Widowed/Separated	35.9	0.89 (0.69-1.14)	1.03 (0.82-1.30)
Schooling, years		p=0.09	p=0.68
0-8	41.9	0.86 (0.70-1.05)	1.01 (0.82-1.25)
9-11	51.8	1.06 (0.82-1.37)	1.12 (0.86-1.42)
≥12	48.8	1.00	1.00
Asset index		p=0.01	p=0.06
Poorest	45.1	1.15 (0.95-1.41)	1.15 (0.96-1.39)
Intermediate	54.7	1.40 (1.14-1.72)	1.28 (1.05-1.26)
Richest	39.1	1.00	1.00
Smoking		p<0.01	p=0.02
No	42.6	1.00	1.00
Yes	62.2	1.46 (1.19-1.79)	1.27 (1.04-1.54)
Excessive alcohol consumption		p=0.03	p=0.12
No	44.6	1.00	1.00
Yes	62.3	1.40 (1.11-1.76)	1.20 (0.95-1.52)
Physical activity		p=0.07	p=0.04
No	48.3	1.20 (0.99-1.45)	1.18 (1.01-1.38)
Yes	40.4	1.00	1.00
Food insecurity		p<0.01	p=0.24
No	40.4	1.00	1.00
Yes	57.9	1.45 (1.20-1.74)	1.13 (0.92-1.38)
Stress, terciles		p=0.02	p=0.54
Least stressed	41.6	1.00	1.00
Intermediates	42.6	1.02 (0.83-1.26)	0.94 (0.77-1.16)
Most stressed	55.2	1.33 (1.07-1.65)	1.07 (0.85-1.35)
Perception of health		p=0.67	p=0.31
Excellent/Very good	50.0	1.00	1.00
Good	44.4	0.89 (0.68-1.16)	0.89 (0.70-1.14)
Regular/Poor	46.1	0.92 (0.71-1.20)	0.82 (0.63-1.06)
Depressive symptoms		p<0.01	p=0.02
No	40.9	1.00	1.00
Yes	58.2	1.42 (1.21-1.68)	1.23 (1.03-1.46)
Health insurance		p=0.23	p=0.28
No	48.9	1.13 (0.93-1.37)	0.91 (0.76-1.08)
Yes	43.4	1.00	1.00
Consultation with physician		p<0.01	p<0.01
No	65.3	1.55 (1.35-1.78)	1.54 (1.36-1.75)
Yes	42.0	1.00	1.00
Community health worker		p=0.81	p=0.47
No/does not know	45.6	0.98 (0.80-1.20)	0.93 (0.75-1.15)
Yes	46.9	1.00	1.00
PHU in the neighborhood		p=0.46	p=0.07
No/does not know	47.3	1.08 (0.88-1.32)	1.19 (0.99-1.43)
Yes	43.9	1.00	1.00

PR: prevalence ratio; 95%CI: 95% confidence interval; PHU: Primary Health Unit.

Adjusted for possible confounders, the following remained associated: young (18 to 39 years), single, intermediate economic status, smoking, depressive symptoms, and not having seen a physician in the past year. Excessive alcohol consumption and stress level lost association; not engaged in physical activity during leisure time gained association (p=0.04), and domicile not registered at a Primary Healthcare Unit remained associated with threshold statistical significance (prevalence ratio - PR=1.19; 95%CI: 0.99-1.43) after adjustments (Table 3).

## DISCUSSION

After investigating the prevalence and factors associated with non-immunization against influenza in risk groups, approximately half of the individuals reported non-immunization in the 12 months prior to the interview. After adjustments, the following were associated with the outcome: young (18 to 39 years), single, intermediate economic level, smoking, no physical activity during leisure time, depressive symptoms, not having seen a physician in the last year, and home not being registered at the Primary Healthcare Unit.

Similar to the present results, regarding the prevalence of non-vaccination, both the Korean study with 1,650 adults in the risk group, conducted in 2009 and 2010, in which 53% had not been immunized,^(8)^ and another study conducted in Massachusetts, United States, in which 56% of adults who responded to the Behavioral Risk Factor Surveillance System (BRFSS) were not vaccinated,^(9)^ demonstrating the difficulty in reaching the goal of 75% vaccination against influenza in the risk population.^(19)^ It is possible to consider the role of the media, which highlights the adverse effects, providing greater perception,^(12)^ and the worldwide anti-vaccine movement, which does not recognize its social benefit, and despite affecting mainly children (the most dense and specific vaccination schedule), has supporters in both the risk group and their caregivers.^(12)^

Corroborating the data of this study, 40% out of 47 thousand diabetics who responded to the BRFSS in 2011,^(20)^ and 58% of Brazilian adults and the elderly with chronic pulmonary respiratory disease who responded to the National Survey on Access, Use, and Promotion of Rational Use of Medicines, in 2013 and 2014, were not vaccinated.^(21)^ In the elderly, a group with a lower prevalence in these results, Brazilian studies pointed out a coverage gap between one quarter and one third ^(22-24)^ relative to the prevalence of 27.4%, according to the 2013 National Health Survey (PNS).^(25)^

The highest likelihood of non-vaccination among young people in this sample contributes to previous statistically significant evidence. In South Korea, the percentage of non-vaccinated individuals was one-third lower when comparing those ≤59 years and ≥60 years, with more than 90% non-compliance in the first group.^(8)^ North-Americans, based on the BRFSS, indicated a 20% to 30% higher probability of an individual between 25 and 49 years not being vaccinated against influenza, as compared to those between 50 and 64 years.^(9)^ In diabetics, the chance of young adults not being vaccinated was twice as high compared to the elderly.^(20)^ In pregnant women, although 1.6% of sample, non-vaccination for more than 80% contributed to the prevalence among young adults. A systematic review pointed out subjective trends for non-vaccination among young people: living alone, not being concerned about influenza, and little social pressure to be vaccinated.^(12)^

Analyses of risk groups showed that single individuals have a higher chance of not being vaccinated compared to married couples.^(9,20,21)^ This allows us to consider that the marital relationship has an important influence on vaccination of the adult population, except after 60 years in studies containing only the elderly,^(21-24)^ either by understanding the benefits of vaccination, the emphasis on the elderly as a Risk Group in the campaign, because there is more time available, or by prioritizing their health at this stage of life at the expense of other tasks previously more valued.

The intermediate economic tercile, even with the vaccine provided by SUS, was associated with the outcome, while the income extremes were more likely to be vaccinated, similar to what was shown in Taiwan, between 1999 and 2012.^(26)^A possible hypothesis would be related to the coverage of public and supplementary health services, where the poorest use the SUS and the Primary Healthcare Unit network, while the richest engage more private plans. Thus, the intermediate tercile may feel helpless for not having coverage in the Primary Healthcare Unit area (gaps in the different neighborhoods and difficult logistics of referral by the user’s address) nor economic conditions for private care.

There was no relation between non-vaccination and schooling in this sample, unlike what was found in studies, both in Campinas (SP) including the elderly,^(22)^ and in Rio Grande with pregnant women.^(27)^ Therefore, schooling is not necessarily linked to the population’s knowledge, based on the benefits of vaccination, considering the educational nature aimed by the campaign of the Ministry of Health.^(3,4,28)^

Even though the harm of smoking is well known, in this sample, 17.5% were smokers. There was a higher prevalence of non-vaccinated among smokers, similar to the American studies including diabetics (52%)^(20)^ or the elderly (63%),^(9)^ and to the 2013 National Health Survey in Brazil, according to which 12.7% of elderly were smokers; smoking was also a factor associated with non-vaccination.^(25)^Smoking may be related to a lower tendency to seek primary prevention, as concluded in a systematic review.^(12)^

Not performing physical activity during leisure time permeated most of the sample, with a statistically significant relation with the outcome. Such data was also present specifically among the elderly.^(22,24)^ The performance of physical activity is related to greater health care, and data suggested individuals with unhealthy habits were less likely to seek primary prevention.^(12)^

A disturbing fact was that 29.7% of interviewees presented with depressive symptoms. The presence of comorbidities increases the prevalence of these symptoms,^(29)^ and home confinement is present in most patients.^(30)^ When assessing the presence of depressive symptoms, a higher probability of non-vaccination was observed in up to 46%, with home confinement and loss of interest as possible justifications.^(24)^

Individuals who had not seen a physician in the last year were the most susceptible to non-vaccination for influenza. This association was also observed in the BRFSS 2009-2010,^(9)^ as well as in the elderly who participated in both the 2013 National Health Survey and the 2010 São Paulo survey.^(24,25)^ Among pregnant women in the city of Rio Grande, the non-vaccination rate in those who did not undergo prenatal care was up to 20 times higher.^(27)^ This reinforces the importance of guidance as to primary prevention, as is highlighted in a systematic review; the probability of non-vaccination is higher in those who interact less with the health system, have fewer medical appointments, and do not have a source of regular care.^(12)^

Those who knew that their household was registered in the Primary Healthcare Unit were less likely to not to be vaccinated, showing a borderline association after adjustments. A study with the elderly reported this relation with statistical significance,^(25)^ expressing the benefits of health systems, management, and implementation of logistics per area of the Primary Healthcare Unit.

As possible limitations, we point out the non-inclusion of all risk groups referenced by the Ministry of Health.^(4)^ Moreover, the outcome was measured by a single question, due to the scope of the survey. The response was self-reported, which, added to the data collection period (April to July), makes recall error possible.

As strengths, we mention the fact that it is a population-based study with a good response rate (90%), including several risk groups. After literature review, it is the only national study with such a combination of data. It has potential for data extrapolation to other municipalities with similar sociocultural contexts, and it is a tool to help identify profiles with failures to achieve the goals of vaccination against influenza.

## CONCLUSION

When investigating prevalence and factors associated with non-vaccination against influenza in risk groups, it was evident that half of the individuals were not vaccinated in the period. Considering the similarity of the influenza-like illness and the current COVID-19 pandemic, which shows higher mortality among the elderly and patients with chronic non-communicable diseases, an increase of primary prevention would reduce the higher mortality rate from influenza, and the use of inpatient and intensive care unit beds, optimizing the response of the installed hospital capacity.

## References

[1] 1. Pôrto Â, Ponte CF. Vacinas e campanhas: as imagens de uma história a ser contada. Hist Cienc Saude-Manguinhos. 2003;10(Suppl 2):725-42. 14965069

[2] 2. Feijó RB, Sáfadi MA. Immunizations: three centuries of success and ongoing challenges. J Pediatr (Rio J). 2006;82(3 Suppl):S1-3. 10.2223/JPED.1497 16826307

[3] 3. Brasil. Ministério da Saúde. Secretaria de Vigilância em Saúde. Programa Nacional de Imunizações 30 anos. Brasília (DF): Ministério da Saúde; 2003 [citado 2020 Mar 28]. Disponível em: http://bvsms.saude.gov.br/bvs/publicacoes/livro_30_anos_pni.pdf

[4] 4. Brasil. Ministério da Saúde. Coordenação Geral do Programa Nacional de Imunizações. Informe técnico: 22ª Campanha Nacional de Vacinação contra a Influenza. Brasília (DF): CGPNI; 2020 [citado 2020 Mar 28]. Disponível em: https://sbim.org.br/images/files/notas-tecnicas/informe-tecnico-ms-campanha-influenza-2020-final.pdf

[5] 5. Brasil. Ministério da Saúde. Sistema de Informações do Programa Nacional de Imunizações (SIPNI). Campanha Nacional de Vacinação Contra Influenza 2020. Brasília (DF): SIPNI; 2020 [citado 2020 Abr 3]. Disponível em: http://sipni-gestao.datasus.gov.br/si-pni-web/faces/relatorio/consolidado/coberturaVacinalCampanhaInfluenza.jsf

[6] 6. Sociedade Brasileira de Imunizações (SBIM). Informe técnico – 09/04/2020: vacinação de rotina durante a pandemia de COVID-19. São Paulo: SBIM; 2020 [citado 2020 Mar 28]. Disponível em: https://sbim.org.br/images/files/notas-tecnicas/nota-tecnica-sbim-vacinacao-rotina-pandemia.pdf

[7] 7. Daufenbach LZ, Duarte EC, Carmo EH, Campagna AS, Santos CA. Morbidade hospitalar por causas relacionadas à influenza em idosos no Brasil, 1992 a 2006. Epidemiol Serv Saúde. 2009;18(1):29-44.

[8] 8. Heo JY, Chang SH, Go MJ, Kim YM, Gu SH, Chun BC. Risk perception, preventive behaviors, and vaccination coverage in the Korean population during the 2009-2010 pandemic influenza A (H1N1): comparison between high-risk group and non-high-risk group. PLoS One. 2013;8(5):e64230. 10.1371/journal.pone.0064230 PMC3656839 23691175

[9] 9. Lu PJ, Gonzalez-Feliciano A, Ding H, Bryan LN, Yankey D, Monsell EA, et al. Influenza A (H1N1) 2009 monovalent and seasonal influenza vaccination among adults 25 to 64 years of age with high-risk conditions--United States, 2010. Am J Infect Control. 2013;41(8):702-9. 10.1016/j.ajic.2012.10.027 PMC5820774 23419613

[10] 10. Brasil. Ministério da Saúde. Secretarias Estaduais de Saúde. Coronavírus (COVID-19); COVID-19 no Brasil. Brasília (DF): Ministério da Saúde; 2020 [citado 2020 Jul 12]. Disponível em: https://susanalitico.saude.gov.br/#/dashboard/

[11] 11. Brasil. Ministério da Saúde. Tecnologia da Informação a Serviço do Sistema único de Saúde (DATASUS). Morbidade hospitalar do SUS - por local de residência - Brasil. Brasília (DF): Ministério da Saúde; 2020 [citado 2020 Abr 6]. Disponível em: http://tabnet.datasus.gov.br/cgi/tabcgi.exe?sih/cnv/nruf.def

[12] 12. Schmid P, Rauber D, Betsch C, Lidolt G, Denker ML. Barriers of influenza vaccination intention and behavior - a systematic review of influenza vaccine hesitancy, 2005 - 2016. PLoS One. 2017;12(1):e0170550. 10.1371/journal.pone.0170550 PMC5268454 28125629

[13] 13. Dumith SC, Paulitsch RG, Carpena MX, Muraro MF, Simões MO, Machado KP, et al. Planejamento e execução de um inquérito populacional de saúde por meio de consórcio de pesquisa multidisplinar. Sci Med. 2018;28(3):ID30407.

[14] 14. World Health Organization (WHO). Global status report on alcohol and health 2014. Geneva: WHO; 2014 [cited 2020 Jul 13]. Available from: https://apps.who.int/iris/bitstream/handle/10665/112736/9789240692763_eng.pdf;jsessionid=1D3CCBC52732B4679780DF859E5E1A59?sequence=1

[15] 15. Craig CL, Marshall AL, Sjöström M, Bauman AE, Booth ML, Ainsworth BE, et al. International physical activity questionnaire: 12-country reliability and validity. Med Sci Sports Exerc. 2003;35(8):1381-95. 10.1249/01.MSS.0000078924.61453.FB 12900694

[16] 16. Segall-Corrêa AM, Marin-León L, Melgar-Quiñonez H, Pérez-Escamilla R. Refinement of the Brazilian household food insecurity measurement scale: recommendation for a 14-item EBIA. Rev Nutr. 2014;27(2):241-51.

[17] 17. Reis RS, Hino AA, Añez CR. Perceived stress scale: reliability and validity study in Brazil. J Health Psychol. 2010;15(1):107-14. 10.1177/1359105309346343 20064889

[18] 18. Santos IS, Tavares BF, Munhoz TN, Almeida LS, Silva NT, Tams BD, et al. Sensibilidade e especificidade do Patient Health Questionnaire-9 (PHQ-9) entre adultos da população geral. Cad Saude Publica. 2013;29(8):1533-43. 10.1590/0102-311x00144612 24005919

[19] 19. Monto AS. Seasonal influenza and vaccination coverage. Vaccine. 2010; 28(Suppl 4):D33-44. Review. 10.1016/j.vaccine.2010.08.027 20713259

[20] 20. Athamneh LN, Sansgiry SS. Influenza vaccination in patients with diabetes: disparities in prevalence between African Americans and Whites. Pharm Pract (Granada). 2014;12(2):410. 10.4321/s1886-36552014000200008 PMC4100953 25035719

[21] 21. Bacurau AG, Francisco PM. Prevalence of influenza vaccination in adults and elderly with chronic respiratory diseases. Cad Saude Publica. 2018; 34(5):e00194717. 10.1590/0102-311x00194717 29846401

[22] 22. Francisco PM, Barros MB, Cordeiro MR. Vacinação contra influenza em idosos: prevalência, fatores associados e motivos da não-adesão em Campinas, São Paulo, Brasil. Cad Saude Publica. 2011;27(3):417-26. 10.1590/s0102-311x2011000300003 21519693

[23] 23. Dip RM, Cabrera MA. Influenza vaccination in non-institutionalized elderly: a population-based study in a medium-sized city in Southern Brazil. Cad Saude Publica. 2010;26(5):1035-44. 10.1590/s0102-311x2010000500025 20563403

[24] 24. Sato AP, Antunes JL, Moura RF, de Andrade FB, Duarte YA, Lebrão ML. Factors associated to vaccination against influenza among elderly in a large Brazilian metropolis. PLoS One. 2015;10(4):e0123840. 10.1371/journal.pone.0123840 PMC4395161 25874953

[25] 25. Bof de Andrade F, Sato AP, Moura RF, Antunes JL. Correlates of influenza vaccine uptake among community-dwelling older adults in Brazil. Hum Vaccin Immunother. 2017;13(1):103-10. 10.1080/21645515.2016.1228501 PMC5287316 27690757

[26] 26. Hsu PS, Lian IB, Chao DY. A population-based propensity score-matched study to assess the impact of repeated vaccination on vaccine effectiveness for influenza-associated hospitalization among the elderly. Clin Interv Aging. 2020;15:301-12. 10.2147/CIA.S238786 PMC7060795 32184579

[27] 27. Mendoza-Sassi RA, Linhares AO, Schroeder FM, Maas NM, Nomiyama S, César JA. Vaccination against influenza among pregnant women in southern Brazil and associated factors. Cien Saude Colet. 2019;24(12):4655-64. 10.1590/1413-812320182412.08382018 31778515

[28] 28. Nakamura EY, Mello LM, Silva AS, Nunes AA. Prevalence of influenza and adherence to the anti-flu vaccination among elderly. Rev Soc Bras Med Trop. 2012;45(6):670-4. 10.1590/s0037-86822012000600003 23295866

[29] 29. Brasil. Ministério da Saúde. Depressão: causas, sintomas, tratamentos, diagnóstico e prevenção. Brasília (DF): Ministério da Saúde; 2020 [citado 2020 Apr 11]. Disponível em: https://www.tjdft.jus.br/informacoes/programas-projetos-e-acoes/pro-vida/dicas-de-saude/pilulas-de-saude/depressao-causas-sintomas-tratamentos-diagnostico-e-prevencao

[30] 30. American Psychiatric Association (APA). Diagnostic and statistical manual of mental disorders (DSM-5). 5th ed. Washington (DC): APA; 2013. p. 991.

