# Supplementary material for: Influenza vaccination coverage in elderly and high-risk adults: characterization of associated factors
Source: Einstein (Sao Paulo). 2021 Jun 28;19:eAO5830. doi: 10.31744/einstein_journal/2021AO5830 (PMC8244927; doi:10.31744/einstein_journal/2021AO5830)
Supplement: Translation

# Translation: Cobertura vacinal de influenza em idosos e adultos de alto risco: caracterização dos fatores associados

**Authors:** Kevin Francisco Durigon Meneghini, Camila Furtado Hood, Letícia Oliveira de Menezes, Raúl Andrés Mendoza-Sassi, Samuel Carvalho Dumith

**Affiliations:** 1 Brasil Faculdade de Medicina, Universidade Federal do Rio Grande, Rio Grande, RS, Brasil.; 2 Brasil Faculdade de Medicina, Universidade Católica de Pelotas, Pelotas, RS, Brasil.

**Keywords:** Vacinas contra influenza, Barreiras ao acesso aos cuidados de saúde, Pesquisa sobre serviços de saúde, Prevenção primária, Saúde Pública, Grupos de risco

## Abstract

**Objetivo:**

Avaliar a prevalência e os fatores associados à não vacinação contra influenza em grupos de risco.

**Métodos:**

Estudo transversal, de base populacional, realizado em Rio Grande (RS). O desfecho foi definido como pertencer aos grupos de risco e não ter se vacinado nos últimos 12 meses. Foram analisadas variáveis demográficas, socioeconômicas, comportamentais e de acesso a serviços de saúde.

**Resultados:**

Participaram 680 indivíduos. A prevalência foi de 46,0% (IC95%: 41,8-50,3), variando de 27,9% (idosos) a 81,8% (gestantes). Adultos jovens, solteiros, de nível econômico intermediário, tabagistas, com sintomas depressivos, que não praticavam atividade física e não consultaram um médico no último ano tiveram maior prevalência de não vacinação.

**Conclusão:**

Metade da amostra não foi vacinada no período. Pela semelhança da síndrome gripal com a doença pelo coronavírus 2019 (COVID-19), aumentar a vacinação minimizaria a mortalidade e a utilização de leitos hospitalares devido à influenza, otimizando a resposta da capacidade hospitalar.

## INTRODUÇÃO

Vacina, do latim “*vaccine*”, significa “derivado de vaca”, remetendo a Edward Jenner, responsável pelos registros no século XVIII, quando observou que camponeses não eram afetados pela varíola quando em contato com vacas infectadas,^(1,2)^ o que foi reconhecido quando verificada a eficácia da inoculação de microrganismo na promoção imunológica, com o menor número de pessoas adoecendo.^(1)^

No Brasil, a primeira campanha de vacinação, idealizada por Osvaldo Cruz, ocorreu em 1904, focando na redução da morbimortalidade da varíola.^(1,2)^ Entretanto, com o medo do desconhecido e o desentendimento da população, desencadeou-se uma resistência popular, culminando na revogação da obrigatoriedade da vacinação e no fracasso da campanha. Isso ficou conhecido como Revolta da Vacina.^(1-3)^

Com melhor entendimento da população sobre os benefícios da vacinação, foi-se, aos poucos, criando o hábito de se vacinar na população e, pelo sucesso alcançado, instituiu-se sua obrigatoriedade, há cerca de 50 anos. Hoje, são obrigatórias 20 vacinas que visam proteger as pessoas de agentes controlados, mas que já foram responsáveis por dizimar populações.^(3)^

A Organização Mundial da Saúde (OMS) é um dos órgãos responsáveis pelo desenvolvimento e pela pesquisa de vacinas, com a intenção de erradicar doenças como varíola, poliomielite e varicela.^(2)^ No Brasil, o órgão responsável pelo calendário vacinal e pela aferição de seu cumprimento é o Ministério da Saúde, via Programa Nacional de Imunizações (PNI), adaptando, dentro do território, a vacinação mais específica para cada região.^(2,3)^ O PNI não atua exclusivamente no Brasil, sendo referência internacional e parceiro de outros países, como o Timor Leste, a Palestina e a Cisjordânia.^(2)^

A primeira campanha da vacinação contra a influenza, em 1999, visou reduzir a morbimortalidade nos idosos devido à sua vulnerabilidade, alcançando cobertura de 87,34%, superando a meta em 17,34 pontos percentuais.^(3)^Após esse sucesso, essa vacina passou a ser oferecida durante o ano todo e integra o calendário vacinal anual.^(3)^ A evolução da meta de vacinação foi aumentando conforme o sucesso da cobertura vacinal e a alteração demográfica demonstrada pelo Instituto Brasileiro de Geografia e Estatística (IBGE) – de 80% em 2008 e 90% em 2017,^(4)^ com cobertura entre 89% e 92%.^(5)^

Em 2020, o Brasil encontrava-se na 22ª campanha de vacinação contra a gripe, porém com um panorama diferente: uma nova pandemia, a doença pelo coronavírus 2019 (COVID-19) deixava o mundo e o Brasil mais atentos aos sintomas gripais.^(4,6)^A cobertura vacinal nesse ano visava, principalmente, facilitar o diagnóstico diferencial entre síndrome gripal comum e COVID-19, permitindo uma investigação e um diagnóstico mais precisos e reduzindo a morbimortalidade da doença.^(4,6)^

Atualmente, priorizam-se os grupos mais vulneráveis à infecção, à imunossupressão provisória ou ao risco de descompensação de doença de base.^(7)^ Dentre eles, além de idosos, encontram-se crianças entre 6 meses e 5 anos; gestantes e puérperas; trabalhadores da área da saúde e do sistema prisional; indígenas; população privada de liberdade; obesos; portadores de doenças crônicas não transmissíveis (DCNT), como respiratória, cardíaca, renal, hepática, neurológica e diabetes; em imunossupressão medicamentosa ou congênita e portadores de trissomias.^(4)^

A cobertura vacinal dos integrantes do grupo de risco, em 2019, superou 84%.^(4)^ Gestantes e crianças apresentaram a menor cobertura (84%), sendo os únicos a não atingirem a meta.^(4)^ Os idosos apresentaram cobertura vacinal em torno de 99%.^(4)^ Outras patologias priorizadas computaram 17% das doses aplicadas na campanha; dentre eles, incluíram-se pacientes portadores de doenças respiratórias crônicas, doenças cardíacas crônicas e diabetes, responsáveis por 81% das doses dentro do grupo.^(4)^

Embora pareça inofensiva, a síndrome gripal pode apresentar sinais e sintomas graves, podendo evoluir para o óbito.^(4,7)^ Dependendo da forma como o patógeno é introduzido à população, consequências devastadoras podem ocorrer, como nas pandemias de 2009, com o H1N1^(8,9)^ e, atualmente, com a COVID-19,^(6)^ cuja estatística oficial publicada até julho de 2020 reportou mortalidade de 3,9% no Brasil.^(10)^ Quando indivíduos hígidos são infectados, eles apresentam maior chance de manifestarem sintomas brandos ou serem assintomáticos; todavia, quando expostos, indivíduos com comorbidades ou imunodeprimidos, apresentam maior probabilidade de manifestação de formas graves da doença.^(7)^

No Brasil, as doenças respiratórias são causas frequentes de internação hospitalar (próximo a 640 mil em 2019), principalmente na população idosa. Mesmo com média de permanência de 6 dias, muitos demandam internações prolongadas, necessidade de leito em unidade de terapia intensiva (UTI) e, por vezes, evoluem ao óbito. A taxa de mortalidade foi de 9,45 por 100 mil habitantes em 2019.^(4,7,11)^

Há pessoas que ficam desprotegidas, incluídas ou não no grupo prioritário,^(4)^ e alguns motivos para a não vacinação são medo, crença que não funciona, percepção do baixo risco de adoecimento, experiência desagradável com imunização prévia, falta de conhecimento e não entendimento do fornecimento pelo Sistema Único de Saúde (SUS).^(12)^ A campanha da vacinação apresenta um caráter educacional, pois introduz conhecimentos gerais sobre a influenza, explica a importância do ato, demonstra possíveis morbimortalidades da patologia e promove a imunização populacional.^(3,4)^

Mesmo com os altos índices de coberturas apresentados pela campanha, há a necessidade de se obterem inquéritos populacionais que contribuam com os dados oficiais, visando identificar fatores associados a não vacinação (socioeconômicos, demográficos, hábitos de vida, comorbidades, proximidade à Unidade Básica de Saúde – UBS, e cadastramento de usuários), com a finalidade de auxiliar gestores em saúde na identificação de características que carecem de maior atenção para a meta de cobertura vacinal ser atingida.^(12)^

## OBJETIVO

Avaliar a prevalência e os fatores associados a não vacinação contra influenza em grupos de risco.

## MÉTODOS

A cidade de Rio Grande localiza-se no estado do Rio Grande do Sul, no extremo sul do Brasil, com aproximadamente 200 mil habitantes, 95% residentes na zona urbana. Sua economia está majoritariamente na atividade portuária. O Índice de Desenvolvimento Humano (IDH) local é 0,744 e o Produto Interno Bruto (PIB) per capita é R$ 36.816,67.^(13)^Ademais, a cidade dispõe de dois hospitais, sendo um deles totalmente voltado ao SUS, e 32 UBS.

Este estudo derivou-se do projeto Saúde da População Rio Grandina, que investigou doenças e fatores de risco em maiores de 18 anos da zona urbana de Rio Grande, sendo excluídos institucionalizados em asilos, hospitais, presídios e com incapacidade física e/ou cognitiva, que impedisse de responder ao questionário. A coleta dos dados ocorreu em 2016. Mais detalhes metodológicos podem ser conferidos em publicação prévia.^(13)^

Foram realizados dois cálculos amostrais. O primeiro verificou a prevalência dos desfechos e o segundo, fatores associados aos desfechos. O processo de amostragem foi constituído de dois estágios. Primeiramente, os setores censitários e, após, os domicílios. Procedeu-se à seleção sistemática, selecionando-se 72 dentre 293 setores censitários elegíveis (25%), dez domicílios por setor em média, selecionando mais setores e menos residências minimizando o efeito de delineamento.

Considerou-se como desfecho não ter recebido a vacina contra a gripe nos últimos 12 meses antecedentes à pesquisa. Consideraram-se no denominador apenas indivíduos que compunham os grupos de risco, de acordo com o Ministério da Saúde:^(4)^ idoso; gestante; portador de DCNT (respiratória, cardíaca, renal, *diabetes mellitus* tipo 1 ou tipo 2 em uso de medicação e obesidade grau III) e indígena. As variáveis independentes incluídas foram: sexo (masculino ou feminino), faixa etária (em anos), cor da pele (branca ou outras), estado civil (casado, solteiro ou viúvo/separado), escolaridade (em anos), índice de bens (em tercis), tabagismo (não fumante e fumante atual), consumo de bebida alcoólica em excesso (sim ou não), atividade física (sim ou não), insegurança alimentar (sim ou não), nível de estresse (em tercis), autopercepção da saúde (excelente/muito boa, boa, regular/ruim), sintomas depressivos (sim ou não), ter plano de saúde (sim ou não), ter consultado um médico nos últimos 12 meses (sim ou não), ter recebido visita de agente de saúde (sim ou não/não sabe), domicílio cadastrado na UBS do bairro (sim ou não/não sabe).

O índice de bens foi obtido pela análise de componentes principais em lista de 11 itens de bens ou características domiciliares. O primeiro componente que explicou 30% da variância em todas variáveis (*eigenvalue* de 3.3) foi extraído e divido em tercis. O consumo excessivo de álcool foi definido como ingerir cinco ou mais doses, para homens, e quatro ou mais doses, para mulheres, no último mês.^(14)^ A atividade física foi mensurada por meio da seção de lazer do Questionário Internacional de Atividade Física^(15)^ e definido como inativo (sem atividade) ou com alguma atividade. Insegurança alimentar (falta de disponibilidade e de acesso ao alimento) foi medida utilizando-se a Escala Brasileira de Insegurança Alimentar^(16)^ Estresse foi medido pela Escala de Estresse Percebido.^(17)^ Sintomas depressivos foram obtidos utilizando-se o *Patient Health Questionnaire-9* (PHQ-9).^(18)^

Concomitante à coleta de dados, realizou-se reprodução parcial do instrumento de 10,5% para controle de qualidade dos dados (índice de Kappa de 0,80). Os questionários foram codificados, revisados e duplamente digitados utilizando-se o programa EpiData, versão 3.1, e os dados foram transferidos posteriormente para o pacote estatístico Stata, versão 11.2, efetuando-se análise exploratória do banco, transformação e categorização das variáveis.

Partiu-se da análise descritiva, com descrição das frequências absolutas e relativas das variáveis. Realizou-se análise bivariada e multivariável, por regressão de Poisson, considerando o efeito do delineamento amostral de 1,3. A análise multivariável foi feita em níveis, controlando-se as variáveis para aquelas do mesmo nível ou de níveis acima. Dividiram-se as variáveis em quatro níveis: primeiro, demográficas e socioeconômicas; segundo, comportamentais; terceiro, de saúde; e quarto, de serviços de saúde. Foi usado teste de Wald para heterogeneidade, mantendo-se no modelo ajustado de variáveis com valor de p≤0,20. O nível de significância estatística foi estabelecido em 5% para testes bicaudais.

O projeto foi aprovado pelo Comitê de Ética em Pesquisa na Área da Saúde da Universidade Federal do Rio Grande (FURG) (CAAE: 52939016.0.0000.5324). Os participantes assinaram o Termo de Consentimento Livre e Esclarecido (TCLE) e, se não alfabetizados, consentiram por meio da impressão digital após leitura em voz alta.

## RESULTADOS

A amostra foi composta de 680 indivíduos (taxa de resposta de 90%). A média de idade foi de 55 anos (desvio padrão – DP =17), variando entre 18 e 96 anos. Na amostra, 59,3% pertenciam ao sexo feminino; 52% tinham de zero a 8 anos de escolaridade; 45,9% tinham 60 anos ou mais; 84,3% referiram cor branca e 44% eram casados. Era fumantes 17,5%, 9% consumiam álcool em excesso, aproximadamente 69% não praticavam atividade física, 33,5% tinham insegurança alimentar, 45% percebiam sua saúde como regular ou ruim, e 29,7% apresentavam sintomas depressivos. Não tinham plano de saúde 47,5%, 17,4% não tinham consultado um médico no último ano e 23,5% referiram ter recebido visita de agente de saúde no domicílio, enquanto 34,9% referiram domicílio registrado em UBS (Tabela 1).

**Tabela 1:** Características da amostra que compõe os grupos de risco de vacinação contra gripe

Variável	n (%)
Sexo	
Masculino	277 (40,7)
Feminino	403 (59,3)
Faixa etária, anos	
18-39	145 (21,3)
40-59	223 (32,8)
≥60	312 (45,9)
Cor da pele	
Branca	573 (84,3)
Outras	107 (15,7)
Estado civil	
Casado	299 (44,0)
Solteiro	211 (31,0)
Viúvo, separado	170 (25,0)
Escolaridade, anos	
0-8	353 (52,0)
9-11	197 (29,0)
≥12	129 (19,0)
Índice de bens	
Mais pobres	246 (36,2)
Intermediários	203 (29,9)
Mais ricos	230 (33,9)
Tabagismo	
Não	561 (82,5)
Sim	119 (17,5)
Consumo de álcool em excesso	
Não	617 (91,0)
Sim	61 (9,0)
Atividade física	
Não	470 (69,3)
Sim	208 (30,7)
Insegurança alimentar	
Não	452 (66,5)
Sim	228 (33,5)
Estresse, tercis	
Menos estressados	245 (36,2)
Intermediários	230 (33,9)
Mais estressados	203 (29,9)
Percepção da saúde	
Excelente/muito boa	104 (15,3)
Boa	270 (39,7)
Regular/ruim	306 (45,0)
Sintomas depressivos	
Não	477 (70,3)
Sim	201 (29,7)
Plano de saúde	
Não	323 (47,5)
Sim	357 (52,5)
Consulta ao médico	
Não	118 (17,4)
Sim	562 (82,6)
Agente de saúde	
Não/não sabe	520 (76,5)
Sim	160 (23,5)
UBS no bairro	
Não/não sabe	442 (65,1)
Sim	237 (34,9)

UBS: Unidade Básica de Saúde.

Na tabela 2, foram discriminados, em ordem decrescente, os grupos de risco, de acordo com o desfecho.

**Tabela 2:** Descrição dos grupos de risco e prevalência de não vacinação

Grupo de risco	n (%)	Prevalência de não vacinação (%)
Gestantes	11 (1,6)	81,8
Isquêmicos (AVC)	38 (5,6)	56,8
Doença respiratória crônica	201 (30,0)	52,2
Cardiopatas	133 (19,5)	47,0
Obesos mórbidos	13 (2,0)	46,2
Hipertensos	366 (53,6)	44,4
Portador de câncer	37 (5,4)	43,2
Diabéticos	90 (13,2)	41,1
Insuficiência renal	34 (5,0)	36,4
Idosos	315 (46,1)	27,9
Total	680 (100,0)	46,0
AVC: acidente vascular cerebral.		

A prevalência de indivíduos do grupo de risco não imunizados nos últimos 12 meses foi de 46,0% (intervalo de confiança de 95% – IC95%: 41,8-50,3), variando de 27,9% (idosos) a 65,3% (não tinham consultado um médico no último ano) e 65,5% para os indivíduos entre 18 e 39 anos (Tabela 3).

**Tabela 3:** Análise bruta e ajustada da prevalência de não vacinação contra gripe para grupos de risco

Variável	Prevalência (%)	Análise bruta RP (IC95%)	Análise ajustada RP (IC95%)
Sexo		p=0,63	p=0,68
Masculino	46,9	1,00	1,00
Feminino	45,4	1,03 (0,90-1,18)	1,03 (0,90-1,18)
Faixa etária, anos		p<0,01	p<0,01
18-39	65,5	2,34 (1,84-3,00)	2,06 (1,57-2,69)
40-59	58,7	2,11 (1,66-2,67)	2,03 (1,60-2,58)
≥60	27,9	1,00	1,00
Cor da pele		p=0,95	p=0,52
Branca	46,1	1,00	1,00
Outras	45,8	0,99 (0,81-1,23)	0,93 (0,75-1,16)
Estado civil		p<0,01	p=0,12
Casado	40,5	1,00	1,00
Solteiro	62,1	1,53 (1,26-1,86)	1,24 (1,01-1,52)
Viúvo, separado	35,9	0,89 (0,69-1,14)	1,03 (0,82-1,30)
Escolaridade, anos		p=0,09	p=0,68
0-8	41,9	0,86 (0,70-1,05)	1,01 (0,82-1,25)
9-11	51,8	1,06 (0,82-1,37)	1,12 (0,86-1,42)
≥12	48,8	1,00	1,00
Índice de bens		p=0,01	p=0,06
Mais pobres	45,1	1,15 (0,95-1,41)	1,15 (0,96-1,39)
Intermediários	54,7	1,40 (1,14-1,72)	1,28 (1,05-1,26)
Mais ricos	39,1	1,00	1,00
Tabagismo		p<0,01	p=0,02
Não	42,6	1,00	1,00
Sim	62,2	1,46 (1,19-1,79)	1,27 (1,04-1,54)
Álcool em excesso		p=0,03	p=0,12
Não	44,6	1,00	1,00
Sim	62,3	1,40 (1,11-1,76)	1,20 (0,95-1,52)
Atividade física		p=0,07	p=0,04
Não	48,3	1,20 (0,99-1,45)	1,18 (1,01-1,38)
Sim	40,4	1,00	1,00
Insegurança alimentar		p<0,01	p=0,24
Não	40,4	1,00	1,00
Sim	57,9	1,45 (1,20-1,74)	1,13 (0,92-1,38)
Estresse, tercis		p=0,02	p=0,54
Menos estressados	41,6	1,00	1,00
Intermediários	42,6	1,02 (0,83-1,26)	0,94 (0,77-1,16)
Mais estressados	55,2	1,33 (1,07-1,65)	1,07 (0,85-1,35)
Percepção da saúde		p=0,67	p=0,31
Excelente/muito boa	50,0	1,00	1,00
Boa	44,4	0,89 (0,68-1,16)	0,89 (0,70-1,14)
Regular/ruim	46,1	0,92 (0,71-1,20)	0,82 (0,63-1,06)
Sintomas depressivos		p<0,01	p=0,02
Não	40,9	1,00	1,00
Sim	58,2	1,42 (1,21-1,68)	1,23 (1,03-1,46)
Plano de saúde		p=0,23	p=0,28
Não	48,9	1,13 (0,93-1,37)	0,91 (0,76-1,08)
Sim	43,4	1,00	1,00
Consulta ao médico		p<0,01	p<0,01
Não	65,3	1,55 (1,35-1,78)	1,54 (1,36-1,75)
Sim	42,0	1,00	1,00
Agente de saúde		p=0,81	p=0,47
Não/não sabe	45,6	0,98 (0,80-1,20)	0,93 (0,75-1,15)
Sim	46,9	1,00	1,00
UBS no bairro		p=0,46	p=0,07
Não/não sabe	47,3	1,08 (0,88-1,32)	1,19 (0,99-1,43)
Sim	43,9	1,00	1,00

RP: razão de prevalência; IC95%: intervalo de 95% de confiança; UBS: Unidade Básica de Saúde.

Ajustado para possíveis confundidores, permaneceram associados: jovens (18 a 39 anos), solteiros, nível econômico intermediário, tabagismo, sintomas depressivos e não ter consultado médico no último ano. Consumo excessivo de álcool e nível de estresse perderam associação; não praticar atividade física no lazer ganhou associação (p=0,04) e domicílio não cadastrado em UBS ficou com associação com significância estatística limiar (razão de prevalência – RP =1,19; IC95%: 0,99-1,43) após ajustes (Tabela 3).

## DISCUSSÃO

Após investigar a prevalência e os fatores associados à não imunização contra a gripe nos grupos de risco, aproximadamente metade dos indivíduos reportou não imunização nos 12 meses prévios à entrevista. Após ajustes, associaram-se ao desfecho: jovens (18 a 39 anos), solteiros, nível econômico intermediário, fumar, não praticar atividade física no lazer, sintomas depressivos, não ter consultado um médico no último ano e domicílio não estar cadastrado na UBS.

Assemelham-se com os presentes resultados, quanto à prevalência de não vacinação, tanto estudo coreano com 1.650 adultos do grupo de risco, realizado em 2009 e 2010, sendo que 53% não tinham sido imunizados,^(8)^ quanto outro realizado em Massachusetts, Estados Unidos, onde 56% dos adultos que responderam ao *Behavioral Risk Factor Surveillance System* (BRFSS) não se vacinaram,^(9)^ demonstrando dificuldade de se alcançar a meta de 75% de vacinação para influenza na população de risco.^(19)^ É possível considerar o papel da mídia, que ressalta os efeitos adversos, proporcionando sua maior percepção,^(12)^e o movimento mundial antivacina, o qual não reconhece seu benefício social, e, apesar de afetar principalmente as crianças (calendário vacinal mais denso e específico), possui adeptos tanto do grupo de risco, quanto de seus cuidadores.^(12)^

Corroborando os dados deste estudo, 40% dentre 47 mil diabéticos que responderam ao BRFSS em 2011,^(20)^ e 58% de adultos e idosos brasileiros com doença respiratória pulmonar crônica que responderam a Pesquisa Nacional sobre o Acesso, Utilização e Promoção do Uso Racional de Medicamentos (PNAUM), em 2013 e 2014, não se vacinaram.^(21)^ Em idosos, grupo com menor prevalência nestes resultados, estudos brasileiros apontaram falha na cobertura entre um quarto e um terço^(22-24)^ em relação à prevalência de 27,4%, segundo a Pesquisa Nacional de Saúde (PNS) de 2013.^(25)^

A maior probabilidade de não vacinação entre jovens desta amostra contribui com evidências prévias estatisticamente significativas. Na Coreia do Sul, a percentagem de não vacinados apresentou-se um terço menor ao comparar aqueles ≤59 anos e ≥60 anos, com não adesão de mais de 90% no primeiro grupo.^(8)^ Estadunidenses, baseados no BRFSS, apontaram probabilidade de 20% a 30% maior de um indivíduo entre 25 e 49 anos não ser vacinado para influenza comparados aqueles entre 50 e 64 anos.^(9)^ Em diabéticos, a chance de adultos jovens não estarem vacinados foi o dobro comparados aos idosos.^(20)^Nas gestantes, apesar de 1,6% da amostra, a não vacinação por mais de 80% contribuiu para a prevalência dentre os adultos jovens. Revisão sistemática apontou tendências subjetivas para não vacinação entre jovens: morar sozinho, não se preocupar com a gripe e pouca pressão social quanto à vacinação.^(12)^

Análises de grupos de risco demonstraram que solteiros têm maior chance de não se vacinarem em relação aos casados.^(9,20,21)^ Isso permite considerar que a relação conjugal exerce importante influência para a vacinação da população adulta, excetuando-se após os 60 anos em trabalhos contendo apenas idosos,^(21-24)^ seja pelo entendimento sobre os benefícios da vacinação, pela ênfase nos idosos como Grupo de Risco na campanha, por haver mais tempo hábil ou por se priorizar sua saúde nessa fase da vida, em detrimento de outros afazeres previamente mais valorizados.

O tercil econômico intermediário, mesmo com a vacina fornecida pelo SUS, mostrou-se associado ao desfecho, enquanto os extremos de renda tiveram maior probabilidade de se vacinarem, semelhante ao apresentado em Taiwan, entre 1999 e 2012.^(26)^ Um possível hipótese seria relacionada a cobertura dos serviços de saúde pública e suplementar, onde os mais pobres utilizam o SUS e a rede da UBS, enquanto os mais ricos contratam mais planos privados; assim, o tercil intermediário pode se sentir desamparado por não possuir abrangência em área da UBS (lacunas nos diferentes bairros e dificuldade de logística de referenciamento pelo endereço do usuário) e nem condição econômica para atendimentos privados.

Não houve relação entre não vacinação e escolaridade nesta amostra, diferentemente do encontrado em estudos, tanto em Campinas (SP), incluindo idosos,^(22)^ quanto em Rio Grande com gestantes.^(27)^ Portanto, a escolaridade não está necessariamente ligada ao conhecimento da população, com base nos benefícios da vacinação, considerando o caráter educacional visado pela campanha do Ministério da Saúde.^(3,4,28)^

Mesmo os malefícios do tabagismo sendo bem difundidos, nesta amostra, 17,5% eram fumantes. Houve maior prevalência de não vacinados entre os tabagistas, semelhante aos estudos americanos incluindo diabéticos (52%)^(20)^ ou idosos (63%)^(9)^ e à PNS de 2013 no Brasil, segundo a qual 12,7% dos idosos eram fumantes; o tabagismo também se mostrou fator associado à não vacinação.^(25)^O tabagismo pode estar relacionado com menor tendência à procura de prevenção primária, como concluído em revisão sistemática.^(12)^

Não realizar atividade física no lazer permeou a maioria da amostra, possuindo relação estatisticamente significativa com o desfecho. Tal dado também se fez presente especificamente entre idosos.^(22,24)^ A realização de atividade física está relacionada com maior cuidado em saúde, e os dados sugerem que os indivíduos que têm hábitos não saudáveis tiveram menor probabilidade de buscar prevenção primária.^(12)^

Dado preocupante foi que 29,7% dos entrevistados apresentaram sintomas depressivos. A presença de comorbidades torna maior a prevalência desses sintomas,^(29)^ e a reclusão domiciliar está presente na maioria dos pacientes.^(30)^ Quando avaliada a presença de sintomas depressivos, observou-se maior probabilidade de não vacinação em até 46%, tendo reclusão domiciliar e perda do interesse como possíveis justificativas.^(24)^

Os indivíduos que não consultaram um médico no último ano foram os mais suscetíveis à não vacinação para gripe. Essa associação foi observada também no BRFSS 2009-2010,^(9)^ bem como em idosos que participaram tanto da PNS de 2013, quanto de inquérito paulista feito em 2010.^(24,25)^ Entre gestantes da cidade de Rio Grande, a não vacinação naquelas que não realizaram pré-natal foi até 20 vezes maior.^(27)^ Reforçando a importância da orientação sobre prevenção primária, como destacado em revisão sistemática, é maior a probabilidade de não vacinação naqueles que interagem menos com o sistema de saúde, realizam menos consultas médicas e não possuem uma fonte de cuidados regular.^(12)^

Aqueles que sabiam que seu domicílio estava cadastrado na UBS apresentaram menor probabilidade de não se vacinar, apresentando associação limítrofe após ajustes. Estudo com idosos reportou essa relação com significância estatística,^(25)^ expressando os benefícios dos sistemas de saúde, de gestão e de implementação de logística por área da UBS.

Como possíveis limitações, aponta-se a não inclusão de todos os grupos de risco referenciados pelo Ministério da Saúde.^(4)^ Ademais, o desfecho foi mensurado por pergunta única, devido à abrangência do inquérito. A resposta foi autorreferida, o que, agregado ao período de coleta de dados (abril a julho), possibilita erro de recordatório.

Como pontos fortes, menciona-se o fato de ser um estudo de base populacional com boa taxa de resposta (90%), incluindo diversos grupos de riscos. Após revisão bibliográfica, é o único estudo nacional a apresentar tal combinação de dados; somado ao seu potencial de extrapolação dos dados para outros municípios com contexto sociocultural semelhante, torna-se um instrumento de auxílio à identificação de perfis nos quais se encontram falhas para atingir as metas de vacinação contra a gripe.

## CONCLUSÃO

Ao investigar prevalência e fatores associados à não vacinação contra influenza em grupos de risco, evidenciou-se que metade dos indivíduos não se vacinou no período. Considerando a similaridade da síndrome gripal com a atual pandemia da COVID-19, que apresenta maior mortalidade entre idosos e portadores de doenças crônicas não transmissíveis, o aumento da prevenção primária reduziria o acréscimo de mortalidade pela influenza, e a utilização de leitos hospitalares e de unidade de terapia intensiva, otimizando a resposta da capacidade hospitalar instalada.
